# Determination of Ontological Well‐Being and Self‐Compassion Levels in Patients with Mastectomy: A Cross‐Sectional Study

**DOI:** 10.1111/scs.70228

**Published:** 2026-04-06

**Authors:** Behice Belkıs Çalışkan, İnci Mercan Annak, Ayşe Kabasakal, Ömür Aktaş, Aslı Emine Büyükkasap, Fatma Yasemin Kutlu

**Affiliations:** ^1^ Department of Nursing, Faculty of Health Sciences Istanbul Beykent University Istanbul Türkiye; ^2^ Department of Surgical Nursing, Faculty of Nursing Gazi University Ankara Türkiye; ^3^ Gazi University Hospital Ankara Türkiye; ^4^ Department of Nursing, Faculty of Health Sciences Acibadem Mehmet Ali Aydinlar University Istanbul Türkiye

**Keywords:** mastectomy, nurse, ontological well‐being, self‐compassion

## Abstract

**Background:**

Individuals who undergo mastectomy encounter many psychosocial problems, and individuals need to be evaluated psychosocially during the surgery process. Although the concepts of ontological well‐being and self‐compassion are new in the literature, they provide important clues for the psychosocial evaluation of individuals.

**Aim:**

This study was planned to determine the ontological well‐being and self‐compassion levels of individuals who have had a mastectomy.

**Methods:**

The study was planned with a descriptive and cross‐sectional design and conducted in the general surgery clinic of a training and research hospital. Data were collected face‐to‐face by reaching 96 individuals who had mastectomy operations. Data were collected using the ‘Socio‐demographic information form, Self‐compassion scale‐short form (SCS‐SF), and Ontological well‐being scale (OWBS)’. Statistical methods were used to evaluate the data.

**Results:**

When the study findings were examined, a statistically positive but weak relationship was found between the ontological well‐being and self‐compassion levels of individuals who had a mastectomy (*r* = 0.363, *p* < 0.05), and a statistically positive but weak relationship was found between all dimensions of OWBS and SCS‐SF (*p* < 0.05).

**Conclusion:**

The significant relationship between the ontological well‐being and self‐compassion levels of individuals who have had a mastectomy and many sociodemographic characteristics reveals that these two psychosocial factors should be evaluated. In addition, the high levels of ontological well‐being and self‐compassion of individuals and the positive effects of these two concepts on each other are variables that should be considered.

**Implication for Practice:**

The study results provide insight into the ontological well‐being and self‐compassion levels of individuals who have had a mastectomy. Nurses can determine patients' coping skills and quality of life by examining how individuals evaluate the past, present, and future within the scope of ontological well‐being and the level of compassion they show to themselves while making psychosocial assessments. With these values determined, a comprehensive nursing plan can contribute to individuals' complete state of psychosocial well‐being.

## 1 Introduction

Breast cancer is the most frequently diagnosed cancer and the leading cause of cancer‐related death among women worldwide, with 2.3 million new cases reported in 2020 [1]. It accounts for approximately 25% of all female cancers [2]. Although its aetiology is multifactorial, involving genetic, hormonal, psychological, environmental, and biochemical factors, advances in early diagnosis and adjuvant treatments have improved survival outcomes and quality of life [3, 4]. Nevertheless, breast cancer treatments may threaten both physical integrity and feminine identity, and mastectomy remains a major source of stress for many women [5].

Surgical management of breast cancer primarily includes breast‐conserving surgery and mastectomy [6, 7]. Following mastectomy, women may experience physical and functional consequences such as scarring, breast asymmetry, changes in nipple sensation, and lymphedema [4, 5, 8, 9, 10]. Beyond these physical changes, the diagnosis and treatment process can be accompanied by substantial psychosocial challenges, including anxiety, depression, anger, uncertainty about the future, helplessness, reduced self‐esteem, fear of death, and body image disturbances [11, 12, 13]. These psychosocial difficulties may persist over time and can influence coping, adaptation, and overall well‐being after surgery.

In recent years, increasing attention has been directed toward psychosocial resources that may support adjustment after mastectomy, particularly self‐compassion [12, 14]. Self‐compassion refers to an individual's compassionate and understanding stance toward oneself in the face of suffering, inadequacy, failure, or distressing experiences [12]. It has been conceptualized as an internal resource that promotes acceptance, emotional balance, and adaptive coping, and it has been consistently associated with higher levels of well‐being [15]. Moreover, self‐compassion may buffer psychological distress, such as depression and anxiety, when individuals encounter adverse events [16]. In women with breast cancer and those undergoing mastectomy‐related treatments, evidence suggests that higher self‐compassion is linked to more positive body‐related experiences and reduced negative body image perceptions [13, 14, 16, 17]. Self‐compassion has also been reported to play a mediating role in alleviating psychological distress, negative body image, and sexuality‐related concerns following mastectomy [18, 19].

Alongside self‐compassion, ontological well‐being has emerged as a relatively new construct in the literature and may offer a broader existential perspective on psychosocial adaptation [20, 21, 22]. Ontological well‐being, also referred to as one's ‘life project’, reflects how individuals evaluate and emotionally experience their past, present, and future in relation to meaning, purpose, and personal responsibility [20, 21]. Şimşek and Kocayörük (2013) described ontological well‐being as a theory‐based construct closely related to an individual's search for meaning and purpose across life domains [21]. Şimşek (2009) further proposed ontological well‐being as an alternative framework to subjective well‐being, emphasizing that emotional evaluations of one's life cannot always be sufficiently captured by traditional well‐being approaches [22]. Importantly, a diagnosis such as breast cancer and the experience of mastectomy may confront individuals with existential concerns, including fear of death and uncertainty, potentially leading to a reassessment of life meaning and future expectations [2, 3]. Therefore, examining ontological well‐being may provide valuable insight into the emotional burden and meaning‐making processes experienced by women following mastectomy [21, 23].

Despite growing evidence regarding the psychosocial importance of self‐compassion, studies focusing on ontological well‐being among individuals who have undergone mastectomy remain limited. Evaluating both constructs together may contribute to a more comprehensive understanding of psychosocial functioning in this population and may inform supportive nursing interventions. Conceptually, self‐compassion may facilitate adaptive meaning‐making by enabling individuals to respond to suffering with kindness rather than self‐criticism, thereby supporting emotional regulation and coping. In turn, this compassionate stance may be associated with more adaptive evaluations of one's life project across past, present, and future, which are central components of ontological well‐being. Accordingly, this study was planned to determine the ontological well‐being and self‐compassion levels of individuals who have had a mastectomy.

## 2 Method

### 2.1 Aim

The aim of this cross‐sectional study was to describe levels of ontological well‐being and self‐compassion among individuals who had undergone mastectomy, and to examine their associations as well as differences according to sociodemographic and clinical characteristics.

### 2.2 Research Design

This study employed a descriptive, cross‐sectional design. The study was conducted between November 2022 and January 2023 in the breast surgery clinic of a training and research hospital, where mastectomy procedures are frequently performed. A cross‐sectional approach was considered appropriate for the exploratory aims of describing ontological well‐being and self‐compassion and examining their associations in individuals who had undergone mastectomy. Data were collected through face‐to‐face interviews by one of the researchers working in the clinic, primarily on the 2nd and 3rd postoperative days.

### 2.3 Setting and Participants

The study was conducted between November 2022 and January 2023 in the breast surgery clinic of a training and research hospital, a high‐volume unit where mastectomy procedures are routinely performed. Participants were recruited consecutively from eligible patients who had undergone mastectomy and were hospitalized in the clinic during the data collection period. Data were collected through face‐to‐face interviews by one of the researchers, primarily on the 2nd and 3rd postoperative days. To enhance clinical interpretability, key clinical characteristics were recorded to contextualize participants' treatment trajectory, including time since diagnosis, surgery date, and history of chemotherapy and radiotherapy. Individuals aged 18 years and older who had undergone mastectomy and voluntarily agreed to participate were included, while those who provided incomplete responses, withdrew at any stage, or had end‐stage breast cancer were excluded. Sample size planning was based on the descriptive and exploratory aims of the study. A priori power analysis was conducted using G*Power 3.1, assuming a medium effect size (Cohen's d = 0.50), a significance level of 0.05 (α = 0.05), and 90% power (1–β = 0.90), which indicated that a minimum of 86 participants would be required to reliably evaluate mean scale scores. A total of 96 participants were included in the study, yielding an achieved power of 1.00. In line with the conceptual basis of the scales and sample‐based structural considerations, the study by Akcan et al. was used as a reference [24]. Written and verbal informed consent was obtained from all participants prior to data collection.

Criteria for inclusion in the study:

- Volunteering to participate in research
- Being over 18 years old
- Having had mastectomy surgery

Exclusion criteria from the study:

- Answering data collection forms incompletely.
- Wanting to withdraw from the study at any stage of the study.
- Having end‐stage breast cancer (Data on the level of self‐compassion, and ontological well‐being of end‐stage breast cancer patients may affect the purpose of the study).

### 2.4 Data Collection

Data were collected using the Individual Characteristics Form, the Ontological Well‐Being Scale (OWBS), and the Self‐Compassion Scale–Short Form (SCS‐SF). Data collection was conducted through face‐to‐face sessions, primarily on postoperative days 2–3, in a quiet area of the ward. Participants who were able to read and write completed the questionnaires independently. For participants who requested assistance (e.g., due to fatigue or low literacy), the researcher read the items aloud and recorded the participants' responses verbatim without providing interpretation or guidance.

### 2.5 Individual Characteristics Form

This form, developed by scanning the literature, consists of questions about the patients' personal, disease‐related attitudes towards the disease [4, 6, 7].

### 2.6 Ontological Well‐Being Scale (OWBS)

Ontological Well‐Being Scale was developed by Şimşek and Kocayörük in 2013 [21]. The scale gave meaningful results and was found to be related to the analyses made with the concepts of psychological well‐being. After validity and reliability studies were carried out, the scale was subjected to explanatory factor analysis. As a result of factor analysis, a 4‐factor structure was deemed appropriate for the scale. These four sub‐dimensions are defined as ‘nothingness’, ‘activation’, ‘regret’ (regression), and ‘hope’. The scale is grouped as ‘Past’, ‘Present’ and ‘Future’. The scale is a 5‐point Likert‐type measurement tool and consists of 24 items. While items 1, 3 and 8 on the scale are scored reversely, the other items are scored as 1, I don't feel it at all; 2, I feel it a little; 3, I feel it intensely; 4, I feel it quite intensely, and 5, I feel it very intensely. The total score obtained from the scale is a minimum of 24 and a maximum of 120 points. As the score obtained from the scale increases, the ontological well‐being levels of the participants increase. The reliability level of OWBS in this study was found to be 0.72 with Cronbach's Alpha (Crα) coefficient [21]. In this study, the alpha value was calculated as 0.913. The alpha values for the scale's sub‐dimensions were calculated as regret 0.845, action 0.782, nothingness 0.899, and hope 0.969.

The OWBS was considered particularly relevant for individuals undergoing mastectomy, as this experience may trigger existential concerns and a re‐evaluation of one's life project across past, present, and future, including future expectations. In this context, ontological well‐being complements self‐compassion by capturing meaning‐oriented and time‐based appraisals of life, thereby providing a broader understanding of psychosocial adjustment following surgery. Although the OWBS has previously been used in various adult samples, its use in individuals who have undergone mastectomy remains limited. In the present study, the OWBS demonstrated high internal consistency, supporting its reliability in this clinical population, and it was significantly associated with self‐compassion in line with theoretical expectations.

### 2.7 Self‐Compassion Scale‐Short Form (SCS‐SF)

SCS‐SF was developed by Neff in 2003 [25]. The “Self‐Compassion Scale Short Form (SCS‐SF)” was developed by Raes, Pommier, Neff, and Van Gucht, considering that it would be more useful in terms of time than the long form [26]. SCS‐SF is a 5‐point Likert type, 11‐item, and unidimensional scale that includes positive and negative factors from the 26‐item Self‐Compassion Scale. The scale's 1st, 4th, 8th, 9th, 10th, and 11th items are reverse coded. The total score obtained from the scale is a minimum of 11 and a maximum of 55 points. As the score obtained from the scale increases, the self‐compassion levels of the participants increase. As a result of the statistical analysis, the Crα coefficient of the entire scale was calculated as 0.75 [27]. In this study, the alpha value was calculated as 0.715.

### 2.8 Data Analysis

Data were analysed using IBM SPSS Statistics version 25. Internal consistency of the OWBS and SCS‐SF was assessed using Cronbach's alpha coefficients. Normality of continuous variables was examined and the data were not normally distributed; therefore, non‐parametric statistical tests were applied. The Mann–Whitney U test was used to compare two independent groups, and the Kruskal–Wallis H test was used to compare three or more independent groups. Spearman's rank correlation coefficient was used to examine the associations between ontological well‐being and self‐compassion scores. A two‐tailed *p* value of < 0.05 was considered statistically significant.

### 2.9 Ethical Consideration

The study was approved by the Beyken University Scientific Research and Publication Ethics Board for Social and Human Sciences (dated 20.12.2021, number 128) and institutional permission was obtained. Participations were informed according to the Helsinki Declaration, and their written and verbal consent was obtained. The individuals who participated in the research were assured that their information would not be disclosed to anyone other than the researcher and were bound to the principle of confidentiality.

## 3 Results

Information regarding the study findings is included in the tables and description areas.

Table 1 summarizes the sociodemographic and clinical characteristics of the participants (*n* = 96). Most patients were married (67.7%), lived in a province (50.0%), and reported an income level equal to their expenses (68.8%). The majority were not working (83.3%) and lived in a nuclear family (85.4%). More than half had a chronic disease (59.4%) and most had children (77.1%). Regarding health status, 38.5% rated their health as ‘neither good nor bad’, while 19.8% rated it as good and 25.0% as very bad. A family history of breast cancer was reported by 30.2% of participants, and most had breastfed (77.1%) and were postmenopausal (83.3%). The mean age was 54.06 years and the mean age at menopause was 45.01 years. In terms of clinical context, 39.6% had undergone surgery in 2021 and 24.0% in 2022 or later. Approximately one‐third had been diagnosed within the last year (34.4%) and 32.3% reported being diagnosed a few months earlier. Most participants had received chemotherapy (81.3%), whereas 42.7% reported receiving radiotherapy. Psychosocially, 65.6% reported family‐related problems during the illness process, 83.3% reported changes in life due to the disease, and most perceived sufficient support from family (79.2%) and friends/environment (72.9%) during the disease process.

**TABLE 1:** Individual characteristics of the patients (*n* = 96).

Variables		n	%
Marital status	Single	31	32.3
	Married	65	67.7
Educational status	Primary school	26	27.1
	University	25	25.5
	High‐school	25	26.5
	Literate	7	7.3
	Middle school	13	13.5
Place of residence	Province	48	50.0
	Town	39	40.6
	Village	9	9.4
Income status	Income is less than expenses	19	19.8
	Income exceeds expenses	11	11.5
	Income equals expenses	66	68.8
Working status	Working	16	16.7
	Not working	80	83.3
Family type	Nuclear family	82	85.4
	Extended family	14	14.5
Chronic disease	Yes	57	59.4
	No	39	40.6
Whether or not you have children	Yes	74	77.1
	No	22	22.9
Health situation	Very good	6	6.3
	Too bad	24	25.0
	It's ok (neither good nor bad)	37	38.5
	Good	19	19.8
	Bad	10	10.4
Family history of breast cancer	There is	29	30.2
	None	67	69.8
Breastfeeding status	Yes	74	77.1
	No	22	22.9
Menopause status	Yes	80	83.3
	No	16	16.7
Surgery date	2019 and before	12	12.5
	year 2020	23	24.0
	year 2021	38	39.6
	2022 and beyond	23	24.0
Time of disease diagnosis	It's been 1 year	33	34.4
	It's been 2 years	26	27.1
	3 years and above	6	6.3
	A few months ago	31	32.3
Chemotherapy history	Yes	78	81.3
	No	18	18.8
Radiotherapy history	Yes	41	42.7
	No	55	57.3
Problems in the family related to the disease	Yes	63	65.6
	No	33	34.4
Changes caused by the disease in life	Yes	80	83.3
	No	16	16.7
Family support during the disease process	Sufficient	76	79.2
	Insufficient	20	20.8
Friend/environment support during the disease process	Sufficient	70	72.9
	Insufficient	26	27.1
	Never happened	16	16.7

Table 2 presents the mean total scores and sub‐dimension scores of the Ontological Well‐Being Scale (OWBS) and the Self‐Compassion Scale–Short Form (SCS‐SF). Table 3 summarizes group comparisons of OWBS and SCS‐SF scores according to participants' sociodemographic and clinical characteristics using the Mann–Whitney *U* and Kruskal–Wallis H tests.

**TABLE 2:** Mean scores of OWBS and SCS‐SF (*n* = 96).

	Min.	Max.	X̄	S.S.
**OWBS**	**24**	**120**	**62.14**	**18.68**
Regret	7	35	20.81	6.95
Take action	5	25	9.36	4.02
Nothingness	6	30	19.05	7.65
Hope	6	30	12.91	7.00
**SCS‐SF**	**11**	**55**	**32.18**	**6.31**

*Note:* Bold values denote statistically significant differences or associations (*p* < 0.05).

**TABLE 3:** Comparison of the individual characteristics with the OWBS and SCS‐SF points of the patients.

Variables		OWBS	SCS‐SF
		Med	Med
Marital status	Single	37.32	39.69
	Married	53.83	52.70
	**MWU**	661.00	764.50
	**p**	0.**007**	0.080
Educational status	Primary school	47.77	61.29
	Licence	53.23	49.88
	High school	46.83	40.87
	Literate	46.00	42.71
	Middle school	45.92	38.77
	**KWH**	0.97	9.63
	**p**	0.914	0.**047**
Place of residence	Province	57.91	49.24
	Town	39.86	47.36
	Village	35.78	49.50
	**KWH**	11.12	0.11
	**p**	0.**004**	0.945
Income status	Income is less than expenses	56.34	55.34
	Income exceeds expenses	46.86	55.27
	Income equals expenses	46.52	45.40
	**KWH**	1.88	2.68
	**p**	0.390	0.261
Working status	Working	60.78	53.28
	Not working	46.04	47.54
	**MWU**	443.50	563.50
	**p**	0.053	0.446
Family type	Nuclear family	49.67	49.57
	Extended family	42.17	42.70
	**MWU**	512.50	520.50
	**p**	0.337	0.374
Chronic disease	Yes	46.98	48.98
	No	50.72	47.79
	**MWU**	1025.00	1084.00
	**p**	0.518	0.835
Whether or not you have children	Yes	49.18	50.05
	No	46.20	43.27
	**MWU**	763.50	699.00
	**p**	0.659	0.310
Health situation	Very good	69.08	60.67
	Too bad	35.27	33.94
	It's ok (neither good nor bad)	47.82	52.09
	Good	70.74	58.05
	Bad	28.15	44.70
	**KWH**	26.217	11.02
	**p**	0.**000**	0.**026**
Family history of breast cancer	There is	52.79	52.12
	None	46.64	46.93
	**MWU**	847.00	866.50
	**p**	0.320	0.396
Breastfeeding status	Yes	51.16	51.11
	No	39.57	39.73
	**MWU**	617.50	621.00
	**p**	0.086	0.088
Menopause status	Yes	47.26	47.69
	No	54.72	52.53
	**MWU**	540.50	575.50
	**p**	0.327	0.521
Surgery date	2019 and before	67.63	56.67
	2020	39.35	43.72
	2021	38.92	41.75
	2022 and beyond	63.50	60.17
	**KWH**	19.34	8.19
	**p**	0.**000**	0.055
Time of disease diagnosis	It's been 1 year	46.24	45.79
	It's been 2 years	39.00	49.40
	3 years and above	73.58	47.67
	A few months ago	54.02	50.79
	**MWU**	9.34	0.57
	**p**	0.**025**	0.903
Chemotherapy history	Yes	45.17	45.83
	No	62.94	60.08
	**MWU**	442.00	493.50
	**p**	0.**015**	0.057
Radiotherapy history	Yes	44.04	48.13
	No	51.83	48.77
	**MWU**	944.50	1112.50
	**p**	0.175	0.910
Problems in the family related to the disease	Yes	39.13	42.64
	No	66.38	59.68
	**MWU**	449.50	670.50
	**p**	0.**000**	0.**004**
Changes caused by the disease in life	Yes	43.28	47.88
	No	74.59	51.59
	**MWU**	222.50	590.50
	**p**	0.**000**	0.622
Family support during the disease process	Yes	52.22	52.95
	No	34.38	31.60
	**MWU**	477.50	422.00
	**p**	0.**011**	0.**002**
Friend/Environment support during the disease process	Yes	52.69	52.84
	No	37.23	36.83
	**MWU**	617.00	606.50
	**p**	0.**016**	0.**011**

*Note:* Bold values denote statistically significant differences or associations (*p* < 0.05).

For OWBS, statistically significant differences were observed by marital status, place of residence, perceived health status, surgery date, time since diagnosis, chemotherapy history, presence of family‐related problems during the illness process, perceived life changes due to the disease, and perceived support from family and friends/environment (*p* < 0.05; Table 3). Higher OWBS scores were observed among married participants compared with single participants, and among those living in a province compared with those living in a village. Participants reporting better perceived health status also had higher OWBS scores. In addition, OWBS scores differed according to clinical characteristics, including surgery date and time since diagnosis, and were higher among those who reported receiving chemotherapy. Participants who reported family‐related problems and those who reported disease‐related life changes also had higher OWBS scores. Overall, these findings suggest that perceived health status, illness‐related experiences, and social support may be important contextual factors associated with ontological well‐being in the early postoperative period.

For SCS‐SF, statistically significant differences were found by educational status, perceived health status, surgery date, presence of family‐related problems during the illness process, and perceived support from family and friends/environment (*p* < 0.05; Table 3). Higher self‐compassion scores were observed among participants with primary school education compared with several other education groups, and among those reporting better perceived health status. Participants reporting sufficient support from family and friends/environment had higher self‐compassion scores than those reporting insufficient support. These findings indicate that self‐compassion may vary across social and clinical contexts and may be linked to perceived support and adjustment‐related experiences following mastectomy.

Table 4 shows the results of Spearman correlation analyses examining the relationship between OWBS (total and sub‐dimensions) and SCS‐SF. A statistically significant positive but weak correlation was found between OWBS total scores and self‐compassion (*r* = 0.363, *p* < 0.05). Significant positive correlations were also observed between self‐compassion and all OWBS sub‐dimensions (*p* < 0.05; Table 4). This pattern suggests that higher self‐compassion tends to co‐occur with more positive evaluations of one's life project, although the strength of these associations is modest.

**TABLE 4:** The Correlation between OWBS and SCS‐SF.

Variables	SCS‐SF
**Ontological Well‐Being Scale (OWBS)**	**0.363** **
Regret	0.203*
Take action	0.230*
Nothingness	0.354**
Hope	0.248*

*Note:* Bold values denote statistically significant differences or associations (*p* < 0.05).

* Correlation is significant at the 0.05 level (2‐tailed).

** Correlation is significant at the 0.01 level (2‐tailed).

A multiple regression model examining the contribution of OWBS sub‐dimensions to self‐compassion was statistically significant (F = 25.992, *p* < 0.001) (Table 5). Regret and nothingness/meaninglessness were positively associated with self‐compassion, whereas activation and hope were negatively associated, although not all predictors reached statistical significance. The model explained 19.1% of the variance in self‐compassion (R^2^ = 0.191), indicating a modest explanatory contribution of ontological well‐being dimensions and suggesting that additional factors may influence self‐compassion in this population.

**TABLE 5:** Regression model for the effect of OWBS level on SCS‐SF.

Dependent variable	Independent variables	Unstandardized variables		Standardized variables	t	p	R	_R_2	F	p
		B	S H	β						
SCS‐SF	(Sabit)	1.840	0.157		11.736	0.000	0.438	0.191	25.992	**0.000**
	Pişmanlık	0.208	0.046	0.262	4.569	0.000				
	Aktivasyon	−0.028	0.050	−0.033	−0.563	0.574				
	Hiçlik/anlamsızlık	0.199	0.038	0.272	5.215	0.000				
	Umut	−0.020	0.043	−0.026	−0.462	0.644				

*Note:* Bold values denote statistically significant differences or associations (*p* < 0.05).

## 4 Discussion

Individuals may experience substantial psychosocial challenges following mastectomy. This study examined ontological well‐being and self‐compassion levels among individuals who had undergone mastectomy and explored how these constructs relate to sociodemographic and clinical characteristics. Overall, the findings indicate that ontological well‐being and self‐compassion are positively associated, although the magnitude of this relationship is modest.

The sample characteristics in the present study were broadly comparable to previous research in breast cancer populations, where many participants were married and had children, and where treatment‐related burdens may shape psychosocial functioning [17, 28]. In this study, several contextual factors were associated with ontological well‐being, including perceived health status, time since diagnosis, chemotherapy history, illness‐related life changes, and perceived social support. Although direct comparisons are limited due to the scarcity of studies focusing specifically on ontological well‐being in individuals after mastectomy, these findings align with evidence indicating that psychosocial well‐being following breast cancer surgery is influenced by multiple clinical and social determinants [28, 29, 30, 31]. Mastectomy may be experienced not only as a physical loss but also as a life event that affects identity, body image, and future orientation, potentially increasing existential concerns and uncertainty [32]. From an ontological perspective, such experiences may influence how individuals evaluate their life project across past, present, and future, including regret, meaninglessness, activation, and hope.

The current findings suggest that ontological well‐being may be shaped by both treatment‐related and interpersonal experiences. For example, participants reporting illness‐related life changes and family‐related problems showed differences in ontological well‐being. This may reflect the complexity of psychosocial adjustment in the early postoperative period, when individuals may simultaneously cope with physical recovery and emotionally process changes in their life roles, relationships, and future expectations. Consistent with the literature, psychosocial burden in breast cancer may be reinforced by perceived stigma, altered body image, and disruptions in social functioning, which can contribute to emotional distress and isolation [32]. Therefore, evaluating ontological well‐being may offer clinically meaningful insight into how individuals interpret and emotionally respond to their broader life circumstances after mastectomy.

Self‐compassion also differed across several participant characteristics, including education level, perceived health status, surgery date, family‐related problems, and perceived social support. These findings are consistent with prior studies highlighting self‐compassion as a protective psychosocial resource associated with better adjustment, body image experiences, and reduced distress in women with breast cancer [16, 18, 33]. Previous evidence suggests that self‐compassion may support adaptation by promoting an empathic and accepting stance toward oneself during periods of vulnerability, which may reduce emotional difficulties related to body changes and illness‐related stressors [34]. Interventional studies further indicate that psychological approaches such as acceptance‐ and mindfulness‐based strategies may improve self‐compassion and related outcomes among women undergoing mastectomy or breast cancer treatment [35, 36]. Taken together, these findings underscore the relevance of self‐compassion as a potentially modifiable resource in postoperative psychosocial care.

Importantly, the present study found a statistically significant positive relationship between self‐compassion and ontological well‐being, as well as positive associations between self‐compassion and all OWBS sub‐dimensions. Although the strength of these associations was weak, the pattern suggests that individuals who respond to suffering with greater self‐kindness may also be more likely to evaluate their life project in a more adaptive and hopeful manner. This interpretation is consistent with theoretical perspectives proposing that self‐compassion enhances emotional safety and reduces harsh self‐judgement, thereby supporting psychological well‐being and adaptive coping [37]. Similar evidence in breast cancer populations has also shown relationships between psychological well‐being and constructs such as mindfulness and adaptive coping resources [38, 39]. Nevertheless, given the cross‐sectional nature of the study, these findings should be interpreted as associations rather than causal effects.

## 5 Implications for Nursing Care

The findings highlight several practical implications for nursing care following mastectomy. First, nurses can incorporate brief psychosocial assessment into routine postoperative care by exploring patients' perceived health status, emotional distress, social support and future‐related concerns (e.g., uncertainty, fear, or hopelessness). Second, patient education can be structured to address common psychosocial needs, including body image concerns, symptom management, daily functioning and strategies for seeking support from family and peers. Third, nurses can provide supportive communication that encourages a compassionate self‐stance, such as normalizing emotional reactions, validating distress, and reducing self‐blame. Fourth, strengthening social support may be particularly relevant; involving family members in discharge education, encouraging supportive family communication, and directing patients to peer support groups or counselling services may facilitate adjustment. Finally, simple self‐compassion–based practices (e.g., brief guided breathing exercises, compassionate self‐talk prompts, or short reflective writing tasks) could be introduced as low‐burden strategies to help patients manage distress during early recovery.

In summary, this study contributes to the literature by highlighting the co‐occurrence of ontological well‐being and self‐compassion in individuals after mastectomy and by identifying contextual factors associated with these psychosocial outcomes. Future longitudinal and multicentre studies are recommended to clarify how these constructs evolve across different phases of treatment and survivorship and to inform targeted nursing interventions.

## 6 Conclusions

This study is the first to determine mastectomy patients' self‐compassion and ontological well‐being levels. The study results show that the self‐compassion and ontological well‐being levels of mastectomy patients affect both the patients' lifestyles and that these parameters have a weak positive relationship with each other. The fact that patients were evaluated for the first time with the ontological well‐being parameter determines how they view the past, present, and future concepts caused by the disease in individuals and how they plan their lives accordingly. In this respect, patients' ontological well‐being levels are affected in every aspect of their lives and well‐being. The concept of self‐compassion is an essential element in mastectomy patients. Psychosocial parameters of patients with high levels of self‐compassion are also positively affected. The current findings obtained from the study show that ontological well‐being and self‐compassion levels provide essential data for the evaluation of the psychosocial problems experienced by mastectomy patients, and interventional practices are recommended to increase these values.

The findings should be interpreted with caution, as the study was conducted in a single center and may not fully represent individuals undergoing mastectomy in different regions or healthcare settings.

### 6.1 Limitations

This study has several limitations that should be considered when interpreting the findings. First, the cross‐sectional design captures associations at a single time point and does not allow causal inferences regarding the relationship between ontological well‐being and self‐compassion. In addition, these psychosocial constructs may vary across different phases of recovery and survivorship. Second, the study was conducted in a single center with a relatively small sample, which may limit the generalizability of the results to other clinical settings or populations. Treatment trajectories and supportive care practices may also differ across institutions, potentially influencing psychosocial outcomes and further limiting generalizability. Finally, the observed association between the main variables was modest, and the regression model explained a limited proportion of the variance in self‐compassion, suggesting that additional unmeasured factors may contribute to these outcomes.

Future research should prioritize longitudinal designs to examine changes in ontological well‐being and self‐compassion over time and to better clarify potential temporal relationships. In addition, interventional studies evaluating psychosocial or nursing‐led programmes (e.g., self‐compassion–based support, psychoeducation, or structured supportive counselling) may help determine whether these constructs can be strengthened and whether such improvements translate into better psychosocial adjustment after mastectomy. Multicentre studies with larger and more diverse samples are also recommended to enhance generalizability.

## Author Contributions

All authors contributed to the conception or design of the study or to the acquisition, analysis, or interpretation of the data. All authors drafted the manuscript, or critically revised the manuscript, and gave final approval of the version that was submitted for publication. All authors agree to be accountable for all aspects of the work, ensuring integrity and accuracy.

## Funding

The authors have nothing to report.

## Conflicts of Interest

The authors declare no conflicts of interest.

## Data Availability

Research data are not shared.
